# Supplementary material for: Realization of higher-order topological lattices on a quantum computer
Source: Nat Commun. 2024 Jul 10;15:5807. doi: 10.1038/s41467-024-49648-5 (PMC11237062; doi:10.1038/s41467-024-49648-5)
Supplement: Supplementary file 1 — Supplementary Information [file 41467_2024_49648_MOESM1_ESM.pdf]

Realization of Higher-Order Topological Lattices on a Quantum Computer (Supplementary Information)

Jin Ming Koh [1,2,*] Tommy Tai [3,4,*] and Ching Hua Lee [4,†]

[1] *Division of Physics, Mathematics and Astronomy, Caltech, Pasadena, California 91125, US*
[2] *A*STAR Quantum Innovation Centre (Q.InC), Institute of High Performance Computing (IHPC), Agency for Science, Technology and Research (A*STAR), 1 Fusionopolis Way, #16-16 Connexis, Singapore 138632, Republic of Singapore*
[3] *Department of Physics, MIT, Cambridge, Massachusetts 02142, US*
[4] *Department of Physics, National University of Singapore, Singapore 117542*

* These authors contributed equally to this work.

† phylch@nus.edu.sg

Square Lattice ($d = 2$)

Config.	v_0^x	v_1^x	v_0^y	v_1^y
C0	1.5	1.5	1.5	−1.5
C2	0.5	1.5	0.3	−0.3
C4	0.5	0.5	0.3	−0.3
	w_0^x	w_1^x	w_0^y	w_1^y
C0	0.5	0.5	0.5	−0.5
C2	1.5	0.5	1.7	−1.7
C4	1.5	1.5	1.7	−1.7

Cubic Lattice ($d = 3$)

Config.	v_{00}^x	v_{10}^x	v_{00}^y	v_{10}^y	v_{01}^x	v_{11}^x	v_{01}^y	v_{11}^y	v_{01}^z	v_{11}^z	v_{10}^z	v_{01}^z
C0	1.8	1.8	1.8	−1.8	1.8	1.8	−1.8	1.8	−1.8	1.8	1.8	−1.8
C2a	0.2	1.8	0.2	−0.2	1.8	1.8	−0.2	0.2	−0.2	0.2	0.2	−0.2
C2b	1.8	1.8	0.2	−0.2	1.8	0.2	−0.2	0.2	−0.2	0.2	0.2	−0.2
C4a	0.2	0.2	0.2	−0.2	0.2	0.2	−0.2	0.2	−0.2	1.8	1.8	−0.2
C4b	0.2	0.2	0.2	−0.2	1.8	0.2	−0.2	0.2	−0.2	1.8	0.2	−0.2
C4c	0.2	1.8	0.2	−0.2	1.8	0.2	−0.2	0.2	−0.2	0.2	0.2	−0.2
C6	0.2	0.2	0.2	−0.2	0.2	0.2	−0.2	1.8	−0.2	0.2	0.2	−0.2
C8	0.2	0.2	0.2	−0.2	0.2	0.2	−0.2	0.2	−0.2	0.2	0.2	−0.2
	w_{00}^x	w_{10}^x	w_{00}^y	w_{10}^y	w_{01}^x	w_{11}^x	w_{01}^y	w_{11}^y	w_{01}^z	w_{11}^z	w_{10}^z	w_{01}^z
C0	0.2	0.2	0.2	−0.2	0.2	0.2	−0.2	0.2	−0.2	0.2	0.2	−0.2
C2a	1.8	0.2	1.8	−1.8	0.2	0.2	−1.8	1.8	−1.8	1.8	1.8	−1.8
C2b	0.2	0.2	1.8	−1.8	0.2	1.8	−1.8	1.8	−1.8	1.8	1.8	−1.8
C4a	1.8	1.8	1.8	−1.8	1.8	1.8	−1.8	1.8	−1.8	0.2	0.2	−1.8
C4b	1.8	1.8	1.8	−1.8	0.2	1.8	−1.8	1.8	−1.8	0.2	1.8	−1.8
C4c	1.8	0.2	1.8	−1.8	0.2	1.8	−1.8	1.8	−1.8	1.8	1.8	−1.8
C6	1.8	1.8	1.8	−1.8	1.8	1.8	−1.8	0.2	−1.8	1.8	1.8	−1.8
C8	1.8	1.8	1.8	−1.8	1.8	1.8	−1.8	1.8	−1.8	1.8	1.8	−1.8

Tesseract Lattice ($d = 4$)

Config.	v_{000}^x	v_{100}^x	v_{000}^y	v_{100}^y	v_{010}^x	v_{110}^x	v_{010}^y	v_{110}^y	v_{010}^z	v_{110}^z	v_{100}^z	v_{010}^z	v_{001}^x	v_{101}^x	v_{011}^x	v_{111}^x
C0	1.8	1.8	1.8	−1.8	−1.8	1.8	1.8	1.8	1.8	1.8	1.8	−1.8	1.8	−1.8	1.8	1.8
C2	0.2	0.2	0.2	−0.2	−0.2	0.2	0.2	0.2	0.2	0.2	0.2	−0.2	0.2	−0.2	0.2	0.2
C4	0.2	0.2	0.2	−0.2	−0.2	0.2	0.2	0.2	0.2	1.8	1.8	−0.2	0.2	−0.2	0.2	0.2
C8	0.2	0.2	0.2	−0.2	−0.2	0.2	0.2	0.2	0.2	0.2	0.2	−0.2	0.2	−0.2	0.2	0.2
C16	0.2	0.2	0.2	−0.2	−0.2	0.2	0.2	0.2	0.2	0.2	0.2	−0.2	0.2	−0.2	0.2	0.2
E	0	2.5	0.5	0	0	0.5	2.5	0.5	0.5	0	2.5	0	0.5	0	2.5	0
	v_{001}^y	v_{101}^y	v_{011}^y	v_{111}^y	v_{001}^z	v_{101}^z	v_{011}^z	v_{111}^z	v_{000}^w	v_{100}^w	v_{010}^w	v_{110}^w	v_{001}^w	v_{101}^w	v_{011}^w	v_{111}^w
C0	1.8	1.8	−1.8	1.8	1.8	−1.8	1.8	1.8	−1.8	1.8	1.8	1.8	1.8	1.8	1.8	−1.8
C2	0.2	0.2	−0.2	0.2	0.2	−0.2	0.2	0.2	−0.2	1.8	1.8	1.8	1.8	1.8	1.8	−1.8
C4	0.2	0.2	−0.2	0.2	0.2	−1.8	0.2	1.8	−0.2	0.2	0.2	1.8	1.8	1.8	1.8	−1.8
C8	0.2	0.2	−0.2	0.2	0.2	−0.2	0.2	0.2	−0.2	0.2	0.2	0.2	1.8	1.8	1.8	−1.8
C16	0.2	0.2	−0.2	0.2	0.2	−0.2	0.2	0.2	−0.2	0.2	0.2	0.2	0.2	0.2	0.2	−0.2
E	0.5	2.5	0	0.5	0	0	2.5	0.5	0	0.5	0.5	0.5	0.5	0.5	0.5	0
	w_{000}^x	w_{100}^x	w_{000}^y	w_{100}^y	w_{010}^x	w_{110}^x	w_{010}^y	w_{110}^y	w_{010}^z	w_{110}^z	w_{100}^z	w_{010}^z	w_{001}^x	w_{101}^x	w_{011}^x	w_{111}^x
C0	0.2	0.2	0.2	−0.2	−0.2	0.2	0.2	0.2	0.2	0.2	0.2	−0.2	0.2	−0.2	0.2	0.2
C2	1.8	1.8	1.8	−1.8	−1.8	1.8	1.8	1.8	1.8	1.8	1.8	−1.8	1.8	−1.8	1.8	1.8
C4	1.8	1.8	1.8	−1.8	−1.8	1.8	1.8	1.8	1.8	0.2	0.2	−1.8	1.8	−1.8	1.8	1.8
C8	1.8	1.8	1.8	−1.8	−1.8	1.8	1.8	1.8	1.8	1.8	1.8	−1.8	1.8	−1.8	1.8	1.8
C16	1.8	1.8	1.8	−1.8	−1.8	1.8	1.8	1.8	1.8	1.8	1.8	−1.8	1.8	−1.8	1.8	1.8
E	2	−0.5	1.5	−2	−2	1.5	−0.5	1.5	1.5	2	−0.5	−2	1.5	−2	−0.5	2
	w_{001}^y	w_{101}^y	w_{011}^y	w_{111}^y	w_{001}^z	w_{101}^z	w_{011}^z	w_{111}^z	w_{000}^w	w_{100}^w	w_{010}^w	w_{110}^w	w_{001}^w	w_{101}^w	w_{011}^w	w_{111}^w
C0	0.2	0.2	−0.2	0.2	0.2	−0.2	0.2	0.2	−0.2	0.2	0.2	0.2	0.2	0.2	0.2	−0.2
C2	1.8	1.8	−1.8	1.8	1.8	−1.8	1.8	1.8	−1.8	0.2	0.2	0.2	0.2	0.2	0.2	−0.2
C4	1.8	1.8	−1.8	1.8	1.8	−0.2	1.8	0.2	−1.8	1.8	1.8	0.2	0.2	0.2	0.2	−0.2
C8	1.8	1.8	−1.8	1.8	1.8	−1.8	1.8	1.8	−1.8	1.8	1.8	1.8	0.2	0.2	0.2	−0.2
C16	1.8	1.8	−1.8	1.8	1.8	−1.8	1.8	1.8	−1.8	1.8	1.8	1.8	1.8	1.8	1.8	−1.8
E	1.5	−0.5	−2	1.5	2	−2	−0.5	1.5	−2	1.5	1.5	1.5	1.5	1.5	1.5	−2

Supplementary Table 1. Values of intra- and inter-cell coupling coefficients, respectively $v^{\alpha}_{\boldsymbol{\pi}(\mathbf{r}_\alpha)}$ and $w^{\alpha}_{\boldsymbol{\pi}(\mathbf{r}_\alpha)}$ along direction $\alpha \in \{x, y, z, w\}$ and at parity $\boldsymbol{\pi}(\mathbf{r}_\alpha) \in \mathbb{Z}_2^{d-1}$ on the remaining axes, used in simulations of the square ($d = 2$), cubic ($d = 3$), and tesseract ($d = 4$) HOT lattices. These coefficients define the HOT lattice Hamiltonians, in particular nearest-neighbour hoppings $u^{\alpha}_{\mathbf{r}} = [1 - \pi(r_\alpha)]v^{\alpha}_{\boldsymbol{\pi}(\mathbf{r}_\alpha)} + \pi(r_\alpha)w^{\alpha}_{\boldsymbol{\pi}(\mathbf{r}_\alpha)}$ for parity function π on sites $\{\mathbf{r}\}$ of the lattice, as described in Eqs. (6) and (7) of the main text. In this table we provide explicit numerical values for clarity; a systematic procedure to assign coefficients for corner-mode configurations on square and cubic lattices is available in Ref. [1], generalizable to higher dimensions.

Lattice	Configuration	Initial States $\|\psi\rangle$
Square ($d = 2$)	C2	$\left(\|1,1\rangle \pm \|L,1\rangle\right)/\sqrt{2}$
	C4	$\left(\|1,1\rangle \pm \|L,1\rangle\right)/\sqrt{2}$
		$\left(\|1,L\rangle \pm \|L,L\rangle\right)/\sqrt{2}$
Cubic ($d = 3$)	C4a	$\left(\|1,1,1\rangle \pm \|1,1,L\rangle + \|1,L,L\rangle\right)/\sqrt{3}$
		$\left(\|1,1,1\rangle \pm \|1,L,1\rangle - \|1,L,L\rangle\right)/\sqrt{3}$
	C4b	$\left(\|1,1,1\rangle \pm \|1,L,1\rangle - \|1,L,L\rangle\right)/\sqrt{3}$
		$\left(\|1,1,1\rangle + \|1,L,L\rangle \pm \|L,1,1\rangle\right)/\sqrt{3}$
	C8	$\left(\|1,1,1\rangle \pm \|1,L,1\rangle \pm \|L,1,1\rangle\right)/\sqrt{3}$
		$\left(\|1,1,L\rangle \pm \|1,L,L\rangle \pm \|L,1,L\rangle\right)/\sqrt{3}$
		$\left(\|1,L,1\rangle - \|L,1,1\rangle \pm \|L,L,1\rangle\right)/\sqrt{3}$
		$\left(\|1,L,L\rangle + \|L,L,1\rangle \pm \|L,L,L\rangle\right)/\sqrt{3}$

Supplementary Table 2. Initial states used in IQPE to probe the existence of corner HOT modes, which are constructed as simple superpositions of corner-localized states. Above, a state of form $|x,y\rangle$ is perfectly localized on the site at spatial coordinates (x,y), and likewise in the three-dimensional case. Lattices are of side length L.

Supplementary Note 1: Further Details on Hamiltonians and Lattice Mappings

A. Single-Particle Lattice

a. Generic Lattices

Here we provide an expanded overview of the mapping procedure employed in our work, applied to a generic d-dimensional n-band model on an arbitrary lattice. To start, we consider

$$\mathcal{H} = \sum_{\mathbf{k}} \mathbf{c}_{\mathbf{k}}^{\dagger} \mathcal{H}(\mathbf{k}) \mathbf{c}_{\mathbf{k}} \tag{1}$$

for an n-band Bloch Hamiltonian $\mathcal{H}(\mathbf{k})$. The momenta $\{\mathbf{k}\}$ summed over depends on the specific lattice geometry. In real space, the Hamiltonian is recast into a more intuitive tight-binding form,

$$\mathcal{H} = \sum_{\mathbf{r}\mathbf{r}'} \sum_{\gamma\gamma'} h_{\mathbf{r}\mathbf{r}'}^{\gamma\gamma'} c_{\mathbf{r}\gamma}^{\dagger} c_{\mathbf{r}'\gamma'}, \tag{2}$$

where we have associated the band degrees of freedom with a sublattice structure γ, and coefficient $h_{\mathbf{r}\mathbf{r}'}^{\gamma\gamma'} = 0$ for $\mathbf{r}' - \mathbf{r}$ outside the range of hoppings present in the model, *i.e.* adjacent sites for a nearest-neighbour model, next-adjacent for next-nearest-neighbour, *etc.* The coordinates $\mathbf{r}, \mathbf{r}'$ run over the sites in the lattice. The general form of the single-particle Hamiltonian above is exactly Eq. (1) of the main text.

We seek a mapping of this lattice Hamiltonian onto a one-dimensional chain. Functionally, we thus require an encoding of the position $\mathbf{r}$ and sublattice γ of the particle in the original d-dimensional lattice into the target one-dimensional system. In the present work, we adopt the approach of representing the particle on the original lattice as d particles on the one-dimensional chain, each of a different species and are thus distinguishable, such that the location of the αth particle on the chain is identified with the coordinate r_α of the original particle on the lattice along the αth spatial axis. The sublattice index γ is carried by every particle on the chain. This dilation of a single particle on the lattice into d particles on the chain provide sufficient degrees of freedom for an exact mapping. Explicitly, this map is given by Eq. (2) of the main text, which we reproduce here,

$$\mathbf{c}_{\mathbf{r}\gamma}^{\dagger} \mapsto \prod_{\alpha=1}^{d} \left[\omega_{r_\alpha \gamma}^{\alpha}\right]^{\dagger}, \qquad \mathbf{c}_{\mathbf{r}\gamma} \mapsto \prod_{\alpha=1}^{d} \omega_{r_\alpha \gamma}^{\alpha}, \tag{3}$$

which implies a mapping of single-body hoppings

$$\mathbf{c}_{\mathbf{r}\gamma}^{\dagger} \mathbf{c}_{\mathbf{r}'\gamma'} \mapsto \prod_{\alpha=1}^{d} \left[\omega_{r_\alpha \gamma}^{\alpha}\right]^{\dagger} \omega_{r'_\alpha \gamma'}^{\alpha}. \tag{4}$$

Thus a single-body hopping on the original lattice is mapped to a simultaneous hopping, or equivalently a d-body interaction, of the d particles on the chain, such that the changes in the coordinates of the particle along the different spatial axes are respectively described. That is, the αth particle on the chain hops to reflect changes in the αth coordinate of the original particle on the lattice. Hopping of the original particle from sublattice γ' to γ on the lattice is reflected by the simultaneous hopping of all d particles on the chain from γ' to γ on the chain.

A relevant question concerns the appropriate statistics of the $\{\omega^\alpha\}$ particles on the chain. Exchange statistics is unimportant in the single-particle context—there is no second particle on the d-dimensional lattice to exchange with. That is, the physics exhibited by the single-particle d-dimensional model is identical whether $\mathbf{c}_{\mathbf{r}\gamma}$ are fermionic or bosonic. Then the simplest choice for quantum simulation is to take $\omega_{x\gamma}^{\alpha}$ to be bosonic, that is, to be commuting for different species, different sites or sublattices,

$$\left[\omega_{x\gamma}^{\alpha}, \omega_{x'\gamma'}^{\alpha}\right] = \left[\left(\omega_{x\gamma}^{\alpha}\right)^{\dagger}, \left(\omega_{x'\gamma'}^{\alpha}\right)^{\dagger}\right] = 0 \quad \text{if} \quad \alpha \neq \alpha' \quad \text{or} \quad x \neq x' \quad \text{or} \quad \gamma \neq \gamma'. \tag{5}$$

Moreover, as the mapping requires that exactly one of each species of particle be present on the chain, one can constrain the ω^α bosons to be hardcore,

$$\left\{\omega_{x\gamma}^{\alpha}, \omega_{x\gamma}^{\alpha}\right\} = \left\{\left(\omega_{x\gamma}^{\alpha}\right)^{\dagger}, \left(\omega_{x\gamma}^{\alpha}\right)^{\dagger}\right\} = 0. \tag{6}$$

Supplementary Eqs. (5) and (6) constitute the canonical mixed commutation relations for hardcore bosons. Lastly, applying this mapping to Supplementary Eq. (2) or equivalently Eq. (1) of the main text gives the chain Hamiltonian

$$\mathcal{H}_{\text{chain}} = \sum_{\mathbf{r}\mathbf{r}'} \sum_{\gamma\gamma'} h_{\mathbf{r}\mathbf{r}'}^{\gamma\gamma'} \prod_{\alpha=1}^{d} \left[\omega_{r_\alpha \gamma}^{\alpha}\right]^{\dagger} \omega_{r'_\alpha \gamma'}^{\alpha}, \tag{7}$$

which corresponds to Eq. (3) of the main text. In particular, in mapping from Supplementary Eq. (2) to Supplementary Eq. (7) the number of (nonzero) terms in the Hamiltonian is exactly conserved; the only change is that single-body hoppings of the c particle is represented as a simultaneous hopping, equivalent interaction, of the d species of ω^α bosons on the chain, whilst retaining the same amplitudes $h_{\mathbf{r}\mathbf{r}'}^{\gamma\gamma'}$.

This mapping does not destroy the number-conserving properties of the Hamiltonian. That is, the original lattice Hamiltonian in Supplementary Eq. (2) possesses a $U(1)$ symmetry and is number-conserving in the c particle. The chain Hamiltonian in Supplementary Eq. (7) possesses a d-fold $U(1)$ symmetry and is number-conserving in each species of the ω^α bosons. In our work, we used this number-conservation symmetry as part of a post-selection error mitigation technique, since detecting other than d particles on the chain is unphysical and is indicative of hardware error. The symmetry is also exploited for performance improvements in the tensor network-aided circuit recompilation scheme, as circuit optimization can be preferentially focused on the physical Fock space sectors of the chain model. We refer readers to the Methods section of the main text for technical implementation details.

To re-emphasize, no assumptions have been made about the lattice geometry or hopping terms—this mapping is straightforwardly general to any single-particle lattice Hamiltonian. Different Hamiltonians are characterized by the set of hopping coefficients $\{h_{\mathbf{r}\mathbf{r}'}^{\gamma\gamma'}\}$ and site coordinates $\{\mathbf{r}\}$. On the chain Hamiltonian, the site locations $\{x\}$ required is the union of site coordinates $\{r_\alpha\}$ of the original lattice along each direction α, and the sublattices $\{\gamma\}$ is identical. In the following subsections we give a few examples to illustrate. Once the chain Hamiltonian is obtained, the remainder of the quantum simulation methodology carries through.

b. *Example—Triangular Lattice with Arbitrary Hoppings*

Here we make no assumptions on the specific hoppings $\{h_{\mathbf{r}\mathbf{r}'}^{\gamma\gamma'}\}$, as in Supplementary Eq. (2), for maximum generality but assume that the model occurs on a 2D regular triangular lattice. Without loss of generality, we take the lattice constant $a = 1$—that is, adjacent sites are spaced a unit distance apart. Then the lattice is defined by site coordinates

$$\mathbf{r} \in \left\{ (m_1 - 1)\hat{\mathbf{x}} + (m_2 - 1)\left(\frac{1}{2}\hat{\mathbf{x}} + \frac{\sqrt{3}}{2}\hat{\mathbf{y}}\right) : m_1 \in [L_1], m_2 \in [L_2] \right\}, \tag{8}$$

where L_1, L_2 are the linear sizes of the lattice and the notation $[n] = \{1, 2, \ldots, n\}$ for $n \in \mathbb{N}$. We have written the above such that the origin $\mathbf{r} = \mathbf{0}$ occurs at $m_1 = m_2 = 1$, and we assume $L_1 \geq 2$ and $L_2 \geq 2$, since otherwise the lattice is trivial along one direction and is a one-dimensional regular chain model. The unique coordinates along each axis ($\hat{\mathbf{x}}$ and $\hat{\mathbf{y}}$ directions) are

$$\{r_x\} = \frac{1}{2}\left[2L_1 + L_2 - 2\right] - \frac{1}{2}, \qquad \{r_y\} = \frac{\sqrt{3}}{2}[L_2] - \frac{\sqrt{3}}{2}, \tag{9}$$

where we have used shorthand notations for set operations $c[n] = \{cx : x \in [n]\}$ and $[n] + c = \{x + c : x \in [n]\}$ for any scalar c and $n \in \mathbb{N}$. Thus the set of sites on the chain required, as in Supplementary Eq. (7), is

$$\{x\} = \{r_x\} \cup \{r_y\} = \left(\frac{1}{2}\left[2L_1 + L_2 - 2\right] - \frac{1}{2}\right) \cup \left(\frac{\sqrt{3}}{2}[L_2] - \frac{\sqrt{3}}{2}\right). \tag{10}$$

The d-body simultaneous hopping or interaction coefficients in the chain Hamiltonian inherit directly from $\{h_{\mathbf{r}\mathbf{r}'}^{\gamma\gamma'}\}$ on the original lattice, as written in Supplementary Eq. (7) and discussed thereafter. The specific parameters $\{h_{\mathbf{r}\mathbf{r}'}^{\gamma\gamma'}\}$ depend, and are set based on, the specific model considered on the triangular lattice. To give a simple illustration, a model comprising only on-site potentials and nearest-neighbour hoppings on the triangular lattice has $h_{\mathbf{r}\mathbf{r}'}^{\gamma\gamma'}$ nonzero only when

$$[\mathbf{r} = \mathbf{r}'] \vee \left[(|r_x - r'_x| = 1) \vee r_y = r'_y\right] \vee \left[\left(|r_x - r'_x| = \frac{1}{2}\right) \vee |r_y - r'_y| = \frac{\sqrt{3}}{2}\right], \tag{11}$$

and all remaining $h_{\mathbf{r}\mathbf{r}'}^{\gamma\gamma'}$ for other $\mathbf{r}, \mathbf{r}'$ pairs are zero. Then these terms correspondingly do not appear in the mapped chain Hamiltonian—for instance there are no simultaneous hopping terms on the chain where a boson is transferred from site x to x' with $|x - x'| > 2$, as that is beyond the range of the model.

c. Example—Kagome Lattice with Arbitrary Hoppings

The Kagome lattice can be viewed as a triangular lattice with sites removed at regular locations (to produce the hexagonal plaquettes). Thus our above discussion in Supplementary Note 1 A b applies. In particular the site coordinates on the lattice prior to the removal of sites are given by Supplementary Eq. (8), and the coordinates of sites on the chain required by the mapping are given by Supplementary Eq. (10).

The removal of sites to produce a Kagome lattice need not be performed explicitly on the lattice or chain structure—that is, $\{\mathbf{r}\}$ and $\{x\}$ can be retained without changes—but one simply takes care in writing the lattice Hamiltonian such that no hoppings occur to and from the unphysical sites that were to be removed (occurring in the middle of the hexagonal plaquettes). This simple constraint manifests in the $\{h^{\gamma\gamma'}_{\mathbf{r}\mathbf{r}'}\}$ hopping coefficients of the lattice, which is inherited by the chain, as described.

d. Example—BBH Model on Square Lattice

As a last example, we illustrate the application of our mapping to a concrete model different from the HOT lattices considered in our main text. In particular we examine the BBH model as introduced in seminal works [2, 3], which is a $2 \times 2 = 4$-band insulator typically placed on a square lattice. The Hamiltonian is written

$$\begin{aligned} \mathcal{H} &= \sum_{\mathbf{k}} \mathbf{c}^\dagger_{\mathbf{k}} \mathcal{H}(\mathbf{k}) \mathbf{c}_{\mathbf{k}}, \\ \mathcal{H}(\mathbf{k}) &= (\beta_x + \lambda_x \cos k_x)\,\Gamma_4 + \lambda_x \sin k_x \Gamma_3 + (\beta_y + \lambda_y \cos k_y)\,\Gamma_2 + \lambda_y \sin k_y \Gamma_1 + \delta\Gamma_0 \end{aligned} \tag{12}$$

for intra- and inter-cell hopping amplitudes $\beta_x, \beta_y, \lambda_x, \lambda_y$ and staggered potential δ of the model, and where

$$\begin{aligned} &\Gamma_0 = \sigma^z \otimes \mathbb{I} = \begin{bmatrix} 1 & & & \\ & 1 & & \\ & & -1 & \\ & & & -1 \end{bmatrix}, \qquad \Gamma_1 = -\sigma^y \otimes \sigma^x, = \begin{bmatrix} & & & i \\ & & i & \\ & -i & & \\ -i & & & \end{bmatrix}, \\ &\Gamma_2 = -\sigma^y \otimes \sigma^y, = \begin{bmatrix} & & & 1 \\ & & -1 & \\ & -1 & & \\ 1 & & & \end{bmatrix}, \qquad \Gamma_3 = -\sigma^y \otimes \sigma^z, = \begin{bmatrix} & & i & \\ & & & -i \\ -i & & & \\ & i & & \end{bmatrix}, \\ &\Gamma_4 = \sigma^x \otimes \mathbb{I} = \begin{bmatrix} & & 1 & \\ & & & 1 \\ 1 & & & \\ & 1 & & \end{bmatrix}, \end{aligned} \tag{13}$$

are products of Pauli matrices acting on the 4 degrees of freedom within a unit cell. Note that we have slightly generalized the Hamiltonian in Supplementary Eq. (12) with respect to the original model discussed in Ref. [2, 3], whose formulation fixes $\beta_x = \beta_y$ and $\lambda_x = \lambda_y$.

The Hamiltonian in Supplementary Eq. (12) can straightforwardly be converted into real space through an inverse Fourier transform, retaining the 4 band degrees of freedom as sublattices, following the standard prescription. However, it is convenient to additionally flatten the sublattice structure by doubling the linear size of the square lattice along each direction, such that each sublattice on each site is assigned to a unique site on the larger lattice; the even/odd parity of the site on the larger lattice along each direction identifies the sublattice it represents on the original lattice. This leads to an equivalent reparametrized real-space Hamiltonian, for example treated in Ref. [4],

$$\begin{aligned} \mathcal{H} = &\sum_{x\,\text{odd}} \sum_{y\,\text{odd}} \left[\beta_x c^\dagger_{(x+1)y} c_{xy} + \beta_y c^\dagger_{x(y+1)} c_{xy} + \delta_1 c^\dagger_{xy} c_{xy}\right] \\ &+ \sum_{x\,\text{even}} \sum_{y\,\text{odd}} \left[\lambda_x c^\dagger_{(x+1)y} c_{xy} + \beta_y c^\dagger_{x(y+1)} c_{xy} + \delta_2 c^\dagger_{xy} c_{xy}\right] \\ &+ \sum_{x\,\text{odd}} \sum_{y\,\text{even}} \left[(-\beta_x) c^\dagger_{(x+1)y} c_{xy} + \lambda_y c^\dagger_{x(y+1)} c_{xy} + \delta_3 c^\dagger_{xy} c_{xy}\right] \\ &+ \sum_{x\,\text{even}} \sum_{y\,\text{even}} \left[(-\lambda_x) c^\dagger_{(x+1)y} c_{xy} + \lambda_y c^\dagger_{x(y+1)} c_{xy} + \delta_4 c^\dagger_{xy} c_{xy}\right] + \text{h.c.}, \end{aligned} \tag{14}$$

where we have allowed for generic on-site potentials $\delta_1, \delta_2, \delta_3, \delta_4$, and all parameters can be taken to be real. Comparing this explicit Hamiltonian to the form in Supplementary Eq. (2), we find the following definition of the general hopping parameters $\{h_{\mathbf{r}\mathbf{r}'}\}$ that specify the model,

$$h_{\mathbf{r}\mathbf{r}'} = \begin{cases} \beta_x \mathbf{1}_x^{(o)} \mathbf{1}_y^{(o)} + \lambda_x \mathbf{1}_x^{(e)} \mathbf{1}_y^{(o)} - \beta_x \mathbf{1}_x^{(o)} \mathbf{1}_y^{(e)} - \lambda_x \mathbf{1}_x^{(e)} \mathbf{1}_y^{(e)} & \mathbf{r}' = \mathbf{r} + \hat{\mathbf{x}} \\ \beta_x \mathbf{1}_x^{(e)} \mathbf{1}_y^{(o)} + \lambda_x \mathbf{1}_x^{(o)} \mathbf{1}_y^{(o)} - \beta_x \mathbf{1}_x^{(e)} \mathbf{1}_y^{(e)} - \lambda_x \mathbf{1}_x^{(o)} \mathbf{1}_y^{(e)} & \mathbf{r}' = \mathbf{r} - \hat{\mathbf{x}} \\ \beta_y \mathbf{1}_y^{(o)} + \lambda_y \mathbf{1}_y^{(e)} & \mathbf{r}' = \mathbf{r} + \hat{\mathbf{y}} \\ \beta_y \mathbf{1}_y^{(e)} + \lambda_y \mathbf{1}_y^{(o)} & \mathbf{r}' = \mathbf{r} - \hat{\mathbf{y}} \\ 0 & \text{all other } \mathbf{r}, \mathbf{r}', \end{cases} \tag{15}$$

where $\mathbf{r} = (x, y)$ and for brevity we have used the indicator function $\mathbf{1}_z^{(o)} = 1 - \mathbf{1}_z^{(e)}$ defined to take unit value when z is odd and is zero otherwise. Taking Supplementary Eq. (14) and following through with the mapping in Supplementary Eq. (3), that is,

$$c_{xy}^\dagger \mapsto \left(\omega_x^1\right)^\dagger \left(\omega_y^2\right)^\dagger = \left(\omega_x^x\right)^\dagger \left(\omega_y^y\right)^\dagger, \qquad c_{xy} \mapsto \omega_x^1 \omega_y^2 = \omega_x^x \omega_y^y, \tag{16}$$

where $\{\omega^1, \omega^2\}$, also written as $\{\omega^x, \omega^y\}$ to make the correspondence between the α index and the direction of the lattice the species represents explicit, are the two species of bosons living on the quantum chain, we find the chain Hamiltonian

$$\begin{aligned} \mathcal{H}_{\text{chain}} = & \sum_{x \text{ odd}} \sum_{y \text{ odd}} \left[\beta_x \left(\omega_{x+1}^x\right)^\dagger \omega_x^x n_y^y + \beta_y \left(\omega_{y+1}^y\right)^\dagger \omega_y^y n_x^x + \delta_1 n_x^x n_y^y \right] \\ & + \sum_{x \text{ even}} \sum_{y \text{ odd}} \left[\lambda_x \left(\omega_{x+1}^x\right)^\dagger \omega_x^x n_y^y + \beta_y \left(\omega_{y+1}^y\right)^\dagger \omega_y^y n_x^x + \delta_2 n_x^x n_y^y \right] \\ & + \sum_{x \text{ odd}} \sum_{y \text{ even}} \left[(-\beta_x) \left(\omega_{x+1}^x\right)^\dagger \omega_x^x n_y^y + \lambda_y \left(\omega_{y+1}^y\right)^\dagger \omega_y^y n_x^x + \delta_3 n_x^x n_y^y \right] \\ & + \sum_{x \text{ even}} \sum_{y \text{ even}} \left[(-\lambda_x) \left(\omega_{x+1}^x\right)^\dagger \omega_x^x n_y^y + \lambda_y \left(\omega_{y+1}^y\right)^\dagger \omega_y^y n_x^x + \delta_4 n_x^x n_y^y \right] + \text{h.c.}. \end{aligned} \tag{17}$$

Above $n_z^\alpha = (w_z^\alpha)^\dagger w_z^\alpha$ is the number operator for species α on site z of the chain. For an $L_x \times L_y$ original square lattice, that is, $\mathbf{r} \in ([L_1] - 1) \times ([L_2] - 1)$, the sites required on the chain is simply $[\max(L_1, L_2)] - 1$. This completes our illustration of the mapping; henceforth the quantum simulation methodology described in our main text can be used to simulate $\mathcal{H}_{\text{chain}}$, in exactly the same fashion as we simulated the HOT lattice models.

B. Interacting Multiple-Particle Lattice

Here we describe a natural generalization of the mapping scheme (as detailed in Supplementary Note 1 A) for lattices hosting p interacting particles ($p > 1$). As before, we consider a fully general d-dimensional lattice of arbitrary geometry. Keeping single-body hopping and multi-body interaction terms completely general, we may write the lattice Hamiltonian in real space

$$\begin{aligned} \mathcal{H} &= \sum_{\mathbf{r}\mathbf{r}'} \sum_{\gamma\gamma'} h_{\mathbf{r}\mathbf{r}'}^{\gamma\gamma'} c_{\mathbf{r}\gamma}^\dagger c_{\mathbf{r}'\gamma'} + \sum_{\mathbf{r}_1\mathbf{r}_1'} \sum_{\mathbf{r}_2\mathbf{r}_2'} \sum_{\gamma_1\gamma_1'} \sum_{\gamma_2\gamma_2'} h_{\mathbf{r}_1\mathbf{r}_2\mathbf{r}_1'\mathbf{r}_2'}^{\gamma_1\gamma_2\gamma_1'\gamma_2'} c_{\mathbf{r}_1\gamma_1}^\dagger c_{\mathbf{r}_2\gamma_2}^\dagger c_{\mathbf{r}_1'\gamma_1'} c_{\mathbf{r}_2'\gamma_2'} + \dots \\ &= \sum_{m=1}^{M} \left[\sum_{\mathbf{r}_1\mathbf{r}_1'} \cdots \sum_{\mathbf{r}_m\mathbf{r}_m'} \sum_{\gamma_1\gamma_1'} \cdots \sum_{\gamma_m\gamma_m'} h_{\mathbf{r}_1\dots\mathbf{r}_m\,\mathbf{r}_1'\dots\mathbf{r}_m'}^{\gamma_1\dots\gamma_m\,\gamma_1'\dots\gamma_m'} \left(\prod_{j=1}^{m} c_{\mathbf{r}_j\gamma_j}^\dagger \right) \left(\prod_{j=1}^{m} c_{\mathbf{r}_j'\gamma_j'} \right) \right]. \end{aligned} \tag{18}$$

Above, in the first line, $h_{\mathbf{r}\mathbf{r}'}^{\gamma\gamma'}$ are single-body hopping coefficients, labelled by the site and sublattice $(\mathbf{r}', \gamma')$ that the hop originates from and the destination $(\mathbf{r}, \gamma)$, just as in Supplementary Eq. (2). We have added $h_{\mathbf{r}_1\mathbf{r}_2\mathbf{r}_1'\mathbf{r}_2'}^{\gamma_1\gamma_2\gamma_1'\gamma_2'}$, which are two-body interaction amplitudes labelled by the sites and sublattices $(\mathbf{r}_1, \mathbf{r}_2, \gamma_1, \gamma_2)$ of the creation operators and $(\mathbf{r}_1', \mathbf{r}_2', \gamma_1', \gamma_2')$ of the annihilation operators in the interaction term; equivalently the interaction can be interpreted as a simultaneous hopping of two particles from $(\mathbf{r}_1', \mathbf{r}_2', \gamma_1', \gamma_2')$ to $(\mathbf{r}_1, \mathbf{r}_2, \gamma_1, \gamma_2)$. In similar fashion there can be higher-body interaction terms in the Hamiltonian—an m-body term is generically labelled by a set of m unprimed $\mathbf{r}$ and γ, and a

set of m primed $\mathbf{r}$ and γ. The hopping and interaction coefficients are set to zero for distances exceeding the range of the model—for example, for a model with nearest-neighbour hoppings and interactions, $h_{\mathbf{rr}'}^{\gamma\gamma'} = 0$ and $h_{\mathbf{r}_1\mathbf{r}_2\mathbf{r}_1'\mathbf{r}_2'}^{\gamma_1\gamma_2\gamma_1'\gamma_2'} = 0$ for non-adjacent primed and unprimed sites; for extensively long-ranged models generically all coefficients are nonzero.

In the second line we have expressed the summation of Hamiltonian terms compactly, arranged by the number of bodies m involved in the term. In particular $m = 1$ for single-body hopping, $m = 2$ for two-body interactions, henceforth. This expression of the Hamiltonian is fully general for arbitrary lattice geometry and hopping/interaction terms present in the model. We suppose the Hamiltonian contains up to M-body terms; to be meaningful, this is understood to be at most the number of particles p on the lattice—m-body terms with $m > p$ have trivial (vanishing) action on a p-particle system. A parent Hamiltonian with $M > p$ can always be simplified by dropping all $m > p$ terms while leaving the physics of a p-particle state exactly invariant.

Similar to before, we map each particle on the d-dimensional lattice onto d distinguishable particles, each of a different species, on the one-dimensional chain. The coordinate of the αth species particle on the chain represents the coordinate of the original particle on the lattice in the αth direction. That is, we map

$$\mathbf{c}_{\mathbf{r}\gamma}^\dagger \mapsto \prod_{\alpha=1}^{d} \left[\omega_{r_\alpha\gamma}^\alpha\right]^\dagger, \qquad \mathbf{c}_{\mathbf{r}\gamma} \mapsto \prod_{\alpha=1}^{d} \omega_{r_\alpha\gamma}^\alpha, \tag{19}$$

which is unchanged from Supplementary Eq. (3). As a refresher, $\left(\omega_{x\gamma}^\alpha\right)^\dagger, \omega_{x\gamma}^\alpha$ are particle operators for species α, acting on site x and sublattice γ on the chain. This implies a mapping of m-body terms

$$\left(\prod_{j=1}^{m} c_{\mathbf{r}_j\gamma_j}^\dagger\right)\left(\prod_{j=1}^{m} c_{\mathbf{r}_j'\gamma_j'}\right) \mapsto \left(\prod_{j=1}^{m}\prod_{\alpha=1}^{d}\left[\omega_{r_{j\alpha}\gamma_j}^\alpha\right]^\dagger\right)\left(\prod_{j=1}^{m}\prod_{\alpha=1}^{d}\left[\omega_{r_{j\alpha}'\gamma_j'}^\alpha\right]\right), \tag{20}$$

where $r_{j\alpha}$ is the αth component of the site coordinate vector $\mathbf{r}_j$. To illustrate, single-body terms are mapped as

$$c_{\mathbf{r}\gamma}^\dagger c_{\mathbf{r}'\gamma'} \mapsto \left(\prod_{\alpha=1}^{d}\left[\omega_{r_\alpha\gamma}^\alpha\right]^\dagger\right)\left(\prod_{\alpha=1}^{d}\left[\omega_{r_\alpha'\gamma'}^\alpha\right]\right), \tag{21}$$

and two-body interaction terms are mapped as

$$c_{\mathbf{r}_1\gamma_1}^\dagger c_{\mathbf{r}_2\gamma_2}^\dagger c_{\mathbf{r}_1'\gamma_1'} c_{\mathbf{r}_2'\gamma_2'} \mapsto \left(\prod_{\alpha=1}^{d}\left[\omega_{r_{1\alpha}\gamma_1}^\alpha\right]^\dagger \cdot \prod_{\alpha=1}^{d}\left[\omega_{r_{2\alpha}\gamma_2}^\alpha\right]^\dagger\right)\left(\prod_{\alpha=1}^{d}\left[\omega_{r_{1\alpha'}\gamma_1'}^\alpha\right] \cdot \prod_{\alpha=1}^{d}\left[\omega_{r_{2\alpha'}\gamma_2'}^\alpha\right]\right). \tag{22}$$

Unlike in the simpler case of a single-particle lattice (as in Supplementary Note 1 A), as there are multiple particles present on the lattice here, one has to take into account particle statistics. That is, the commutation relations present in the $c_{\mathbf{r}\gamma}$ operators on the lattice translate into equivalent relations that the $w_{x\gamma}^\alpha$ operators on the chain should satisfy. In carrying through the quantum simulation, ultimately $w_{x\gamma}^\alpha$ are represented as spin operators on the qubits representing the chain sites, which satisfy the same commutation relations; these operators are naturally defined once a correspondence between the states of the qubits and states of the quantum chain is chosen. In more detail, for bosonic particles on the lattice,

$$\left[c_{\mathbf{r}\gamma}, c_{\mathbf{r}'\gamma'}\right] = 0, \qquad \left[c_{\mathbf{r}\gamma}, c_{\mathbf{r}'\gamma'}^\dagger\right] = \delta_{\mathbf{rr}'}\delta_{\gamma\gamma'}, \tag{23}$$

which translates into

$$\left[\prod_{\alpha=1}^{d}\omega_{r_\alpha\gamma}^\alpha, \prod_{\alpha=1}^{d}\omega_{r_\alpha'\gamma'}^\alpha\right] = 0, \qquad \left[\prod_{\alpha=1}^{d}\omega_{r_\alpha\gamma}^\alpha, \prod_{\alpha=1}^{d}\left(\omega_{r_\alpha'\gamma'}^\alpha\right)^\dagger\right] = \delta_{\mathbf{rr}'}\delta_{\gamma\gamma'}. \tag{24}$$

The above conditions are satisfied for bosonic particles on the chain, that is,

$$\left[\omega_{x\gamma}^\alpha, \omega_{x'\gamma'}^{\alpha'}\right] = 0, \qquad \left[\omega_{x\gamma}^\alpha, \left(\omega_{x'\gamma'}^{\alpha'}\right)^\dagger\right] = \delta_{xx'}\delta_{\gamma\gamma'}. \tag{25}$$

For fermionic particles on the lattice, the commutators in Supplementary Eqs. (23) and (24) above are replaced by anti-commutators, as follows from the standard canonical commutation algebra defining particle statistics. Likewise one then seeks $\{w_{x\gamma}^\alpha\}$ that satisfy the anti-commuting version of Supplementary Eq. (24). Ideally, one desires that these constraints on $\{w_{x\gamma}^\alpha\}$ be simplified to a convenient form, similar to Supplementary Eq. (25) for bosonic particles,

but there is a subtlety in the present fermionic case. In particular, in even number of dimensions d of the lattice, fixing Supplementary Eq. (25) but with anti-commutators, thus fixing fermionic particles on the chain, does not produce a system that satisfies the anti-commuting Supplementary Eq. (24)—for there is an even number d of $w_{x\gamma}^{\alpha}$ to exchange and the incurred negative signs cancel to yield an overall commuting relation (*i.e.* bosonic statistics). A convenient solution is simply to promote the lattice to one dimension higher, that is, to $d+1$, with trivial physics (*i.e.* identity transformation under dynamics) in the added direction. Since $d+1$ is odd, this issue is sidestepped, and the mapping carries through.

Explicitly, applying the mapping to from Supplementary Eq. (18), the quantum chain Hamiltonian is

$$\mathcal{H}_{\text{chain}} = \sum_{m=1}^{M} \left[\sum_{\mathbf{r}_1 \mathbf{r}_1'} \cdots \sum_{\mathbf{r}_m \mathbf{r}_m'} \sum_{\gamma_1 \gamma_1'} \cdots \sum_{\gamma_m \gamma_m'} h_{\mathbf{r}_1 \ldots \mathbf{r}_m \, \mathbf{r}_1' \ldots \mathbf{r}_m'}^{\gamma_1 \ldots \gamma_m \, \gamma_1' \ldots \gamma_m'} \left(\prod_{j=1}^{m} \prod_{\alpha=1}^{d} \left[\omega_{r_{j\alpha} \gamma_j}^{\alpha} \right]^{\dagger} \right) \left(\prod_{j=1}^{m} \prod_{\alpha=1}^{d} \omega_{r_{j\alpha}' \gamma_j'}^{\alpha} \right) \right]. \tag{26}$$

Just as in Supplementary Note 1 A, this prescription is general to any lattice geometry and any composition of on-site potential, single-body hopping, and interaction terms; the specific lattice geometry to be considered is specified by the sites $\{\mathbf{r}\}$ of the lattice, and the specific model is specified by the set of parameters $\{h_{\mathbf{r}_1 \ldots \mathbf{r}_m \, \mathbf{r}_1' \ldots \mathbf{r}_m'}^{\gamma_1 \ldots \gamma_m \, \gamma_1' \ldots \gamma_m'}\}$ of the Hamiltonian. The same description of the site coordinates $\{x\}$ required on the chain from Supplementary Note 1 A applies, namely, that $\{x\}$ is the union of site coordinates $\{r_\alpha\}$ along each direction α of the lattice.

Compared to the case of the single-particle lattice in Supplementary Note 1 A, the expense demanded by the mapping for a multiple-particle lattice manifests in two ways—the chain Hamiltonian $\mathcal{H}_{\text{chain}}$ is more complex and contains interaction terms involving a larger number of bodies, and as up to p particles participate on the lattice, up to p sets of the d species of particles on the chain are required to be present, translating to a larger local Hilbert space dimension on each site of the chain (as each species can have occupation number up to p on a site).

After the mapping is performed, the quantum simulation methodology described in our main text or other viable methods can be used to simulate $\mathcal{H}_{\text{chain}}$ on quantum platforms. The results (*i.e.* measured data) are translated through the mapping to be interpreted on the lattice, in similar fashion as our work on HOT lattices. The demonstration of quantum simulation of interacting multi-particle lattices through this approach is outside the scope of the present work but is a promising future direction.

[1] L. Li, M. Umer, and J. Gong, Direct prediction of corner state configurations from edge winding numbers in two- and three-dimensional chiral-symmetric lattice systems, Phys. Rev. B **98**, 205422 (2018).
[2] W. A. Benalcazar, B. A. Bernevig, and T. L. Hughes, Quantized electric multipole insulators, Science **357**, 61 (2017).
[3] W. A. Benalcazar, B. A. Bernevig, and T. L. Hughes, Electric multipole moments, topological multipole moment pumping, and chiral hinge states in crystalline insulators, Phys. Rev. B **96**, 245115 (2017).
[4] Y. Fang and J. Cano, Symmetry indicators in commensurate magnetic flux, Phys. Rev. B **107**, 245108 (2023).
